# Co-expression of Cancer Stem Cell Markers OCT4 and NANOG Predicts Poor Prognosis in Renal Cell Carcinomas

**DOI:** 10.1038/s41598-018-30168-4

**Published:** 2018-08-06

**Authors:** Arezoo Rasti, Mitra Mehrazma, Zahra Madjd, Maryam Abolhasani, Leili Saeednejad Zanjani, Mojgan Asgari

**Affiliations:** 1 Oncopathology Research Centre, Iran University of medical Sciences (IUMS), Tehran, Iran; 2 Hasheminejad Kidney Center, Iran University of Medical Sciences, (IUMS), Tehran, Iran; 3 Department of Molecular Medicine, Faculty of Advanced Technologies in Medicine, Iran University of Medical Sciences, Tehran, Iran

## Abstract

Many renal cancer patients experience disease recurrence after combined treatments or immunotherapy due to permanence of cancer stem cells (CSCs). This study was conducted to evaluate the expression patterns and clinical significance of octamer-binding transcription factor 4 (OCT4) and NANOG as the key stem cell factors in renal cell carcinoma (RCC). A total of 186 RCC tissues were immunostained on a tissue microarray (TMA) for the putative CSC markers OCT4 and NANOG. Subsequently, the correlation among the expression of these markers, the clinicopathological variables and survival outcomes were determined. OCT4 and NANOG were expressed in both the nucleus and the cytoplasm of RCC cells. Coexpression of OCT4 and NANOG in renal cancer was significantly associated with RCC subtypes. A significant association was found among nuclear coexpression of OCT4 and NANOG, worse PFS in RCC, and the clear cell renal cell carcinomas (ccRCC) subtype. The OCT4-nuclear ^*high*^/NANOG-nuclear ^*high*^ phenotype in RCC and ccRCC subtype indicated aggressive tumor behavior and predicted a worse clinical outcome, which may be a useful biomarker to identify patients at high risk of postoperative recurrence and metastasis. Cytoplasmic expression of NANOG could be considered as a novel independent prognostic predictor in patients with renal cancer.

## Introduction

Renal cell carcinoma (RCC) is the most common malignant neoplasm of the kidney and is one of the most lethal genitourinary malignancies, with a 5-year survival rate of 67%^1^. RCC is divided into several histological subtypes including ccRCC, (the most common type) (70%), papillary (pRCC) (10–15%), and chromophobe (chRCC) (5%)^2^.

To date, the existence of CSCs has been demonstrated in a wide variety of malignancies including RCC, breast cancer, leukemia, glioblastoma, and liver carcinoma^3–6^. Based on the CSCs hypothesis, tumor initiation, maintenance, and progression are driven by the unique characteristics of CSCs including multi-lineage differentiation, self-renewal, high expression of stemness genes, and drug resistance^3^.

It is generally proposed that CSCs originate either from progenitor cells that have acquired the ability to self-renew, or adult stem cells that have lost control of proliferation. However, several studies support the theory that CSCs arise from differentiated tumor cells that have undergone a process of dedifferentiation to become more stem-like^4^. Moreover, it seems that the key regulators in ESC also contribute to the pathogenesis of cancers by modulating the differentiation and self-renewal of CSCs^5^.

Octamer-binding transcription factor 4 (OCT4) and NANOG are key regulatory genes that maintain the pluripotency and self-renewal properties of normal stem cells and ESCs^6^. They induce the expression of each other, regulate cancer progression, and are biomarkers of CSCs^7–11^. OCT4 belongs to the POU (Pit-Oct-Unc) transcriptional factor family and plays a key role in stem cell differentiation and pluripotency by determining the fate of embryonic stem cells^12^. The expression of OCT4 has further been shown in human breast cancer stem-like cells, implicating its involvement in tumorigenesis and self-renewal by activating its downstream target genes, such as NANOG and SOX2^13^. NANOG, a downstream target of OCT4, contributes to cell fate determination of the pluripotent inner cell mass during embryonic development^14^. It promotes CSC characteristics in prostate cancer and regulates self-renewal of CSC in human hepatocellular carcinoma (HCC)^5^. Several lines of evidence have suggested that expression of OCT4 and NANOG is closely related to tumorigenesis, distant recurrence, and tumor metastasis after treatment^15–19^. Moreover, coexpression of OCT4 and NANOG is a strong independent predictor of unfavorable outcome and tumor recurrence in HCC patients and is associated with enhanced lung cancer malignancy and pancreatic carcinogenesis^16,17,20^.

There is growing evidence of correlation and crosstalk between tumor progression, metastasis, and stemness pathways; however, the functional and mechanistic significance of the overexpressed stem cell markers in cancer is still obscured and needs to be further clarified^20^. Prior studies have suggested that OCT4 and NANOG have a key role of tumorigenesis and prognosis of cancer. However, the prognostic significance of OCT4 and NANOG is not clearly defined in renal cancers. In this study, for the first time, we examined the expression patterns and clinical significance of both OCT4 and NANOG as CSC markers in RCC and its subtypes using tissue microarray (TMA) based immunohistochemistry (IHC) technique.

## Materials and Methods

### Patients’ Characteristics and Tissue Collection

RCC tissue samples were collected from Hasheminejad Kidney Center, a major university-based referral urology-nephrology center in Tehran, Iran, from 2007 to 2015. Patients who had undergone radical nephrectomy and had no history of preoperative hormone or radiation therapy were included in the current study. Based on the pathology findings and case records, the patient samples were categorized into 3 groups: ccRCC (n = 126), pRCC (n = 31), and chRCC (n = 29).The specimens were embedded in paraffin using a routine pathologic tissue processing technique. Medical records were retrieved to obtain the following clinicopathological parameters: tumor size, metastasis to regional lymph node and renal vein (RVI), microvasulcar invasion (MVI), the Gerota’s fascia, adrenal gland, peripheral fat, and renal pelvis involvements. In addition, pathologic tumor stage was defined according to the pTNM Classification for Renal Cell Carcinoma, 2013^21^. Data of patients were kept fully de-identified in all steps. This study was approved by the Research Ethics Committee of Iran University of Medical Sciences.

### Construction of Tissue Microarrays (TMAs)

Renal tissue TMAs were prepared as described previously^22^. TMA blocks were constructed in 3 copies, each containing one sample from different regions of the tumor.

### Immunohistochemistry (IHC) Staining

The expression of OCT4 and NANOG was immunohistochemically evaluated using the protocol as described previously^22–26^. Briefly, sequential TMA sections were dewaxed at 60 °C for 20 minutes, rehydrated in xylenes, and underwent graded ethanol treatment. Antigen was retrieved by autoclaving tissue sections for 10 minutes in sodium citrate buffer (pH 6.0). Endogenous peroxidase and nonreactive staining were blocked by 3% H_2_O_2_ for 20 minutes at room temperature. Then, the tissue sections were incubated overnight at 4 °C with the following antibody dilutions: anti-OCT4 antibody (ab18976; Abcam, UK) using a 1:100 dilution, and anti-NANOG antibody (ab109250; Abcam, UK) using a 1:150 dilution. After 3 washes in Tris-buffered saline (TBS), sections were incubated with anti-rabbit/anti-mouse envision (Dako, Denmark), as the secondary antibody, for 15 minutes. TMA slides were treated with 3,3′-diaminobenzidine (DAB, Dako) substrate as a chromogen for 10 minutes at room temperature. Sections were lightly counterstained with hematoxylin, dehydrated in alcohol, cleared with xylenes, and mounted. For negative controls, the primary antibody step was replaced with TBS, and only the secondary antibody was used. Human seminoma tissues were used as a positive control for OCT4 and NANOG staining.

### Evaluation of Immunostaining

Two investigators (MA and AR), who had no previous knowledge of clinical and pathological parameters of the patients, examined the immunostained tissue arrays using a semi-quantitative scoring system in a coded manner. In difficult cases, scoring was confirmed by 2 observers and a consensus was achieved.

### Scoring System

Immunostaining of OCT4 and NANOG were performed as described previously^22^. The intensities of OCT4 and NANOG staining were scored on a 4-point scale, ranging from absent to strong (0 = absent, 1 = weak, 2 = moderate, and 3 = strong), without previous knowledge of clinical and pathologic parameters. The overall score was obtained by H-score (Histochemical score) for each case by multiplying the intensity of staining by the percentage of positive cells, then, a final score of 0 to 300 was given to each core^27^. Three cut-off points (200, 160, and 200) were selected based on the median H-scores to categorize the samples as high or low nuclear OCT4 and nuclear or cytoplasmic NANOG expressions, respectively. Cytoplasmic OCT4 expression was not categorized because the calculated median of H-score was 300.

### Statistical Analysis

All data were analyzed using IBM Corp. (Released 2011. IBM SPSS Statistics for Windows, Version 20.0. Armonk, NY: IBM Corp.). We reported the categorical data by N, valid percent by %, and quantitative data as mean (SD) and median (Q1, Q3).

Nuclear and cytoplasmic OCT4 and NANOG expressions in ccRCC, pRCC, and chRCC tissues were compared using Kruskal-Wallis test. Mann-Whitney U test was used for pairwise comparison between groups based on Bonferroni adjustment. Moreover, Pearson’s chi square test and Spearman’s correlation coefficient were used to analyze the association between OCT4 and NANOG expressions and clinicopathological parameters, as appropriate. Statistical significance was set at *p* < 0.05.

Disease specific survival (DSS) was measured from the date of nephrectomy to the date of death by RCC. Progression-free survival (PFS) was defined as the interval between the primary surgery and the last follow-up visit if the patient showed no evidence of disease, recurrence, or metastasis of RCC, or disease related death. DSS and PFS curves were calculated using the Kaplan-Meier method. Log-rank test was used to compare the estimated curves between groups. The Cox proportional hazards model was used to determine which variables influenced DSS and PFS. Variables that significantly impacted survival in univariate analysis were included in the multivariable (adjusted) analysis, and significance level was set at *p* < 0.05.

### Ethical Approval

All procedures performed in studies involving human participants were in accordance with the ethical standards of the institutional and/or national research committee and with the 1964 Helsinki declaration, and its later amendments or comparable ethical standards. This research study was approved by the Research Ethics Committee of Iran University of Medical Sciences.

### Informed Consent

Informed consent was obtained from all individual participants in the study.

## Results

### Patient Characteristics

Of the 186 RCC samples that were included in the present study, 126 (67.7%) were ccRCC, 31 (16.7%) pRCC, and 29 (15.6%) chRCC. A total of 130 (69.9%) samples were from male and 56 (30.1%) from female patients. Overall, the mean age of the population was 55.2 (SD = 13.2) years (27–82). Tumor size was categorized into 4 groups: ≤4 cm, 4–7 cm, 7–10 cm, and ≥10 cm^28^. Median tumor size was 7 cm (5, 10), (1.5–21 cm). A total of 56 (30.1%) specimens were stage I, 19 (10.3%) stage II, 99 (53.2%) stage III, and 12 (6.4%) stage IV. Moreover, 9 (4.8%) specimens were grade I (having a low-nuclear grade), 85 (45.7%) grade II, 57 (30.6%) grade III, and 6 (3.2%) had high-nuclear grade (grade IV). Regional lymph node involvement was found in 6 cases (3.2%), whereas 149 cases (80.1%) had no regional lymph node involvement, and in 29 cases (15.6%) no lymph node was dissected during surgery. Of the cases, 38 (20.4%) had MVI, 96 (51.6%) renal sinus involvement, and 65 (34.9%) tumoral necrosis. Other reported involvements were as follow: renal vein, 11 cases (5.9%); adrenal gland, 5 cases (2.7%); Gerota’s fascia, 6 cases (3.2%); renal pelvis, 12 cases (6.5%); and peripheral fat, 33 cases (17.7%).

Patients with RCC were evaluated for survival analysis, and it was found that according to the reverse K-M method, the median follow-up time of the surviving patients in PFS was 56.0 months; 95% CI, 51.24–60.75 while the mean follow-up time for DSS was 96.36 months; 95% CI, 89.35–103.36.

During the follow-up, disease-related death occurred in 25 patients (13.4%). Of the patients, 34 (18.2%) had history of metastasis, while recurrence was observed only in 6 patients (3.2%). Of the 186 patients, 96 (51.3%) had no history of recurrence, metastasis, or disease-related death.

### Comparison of OCT4 and NANOG Expressions in RCC Subtypes

**Figure 1:**
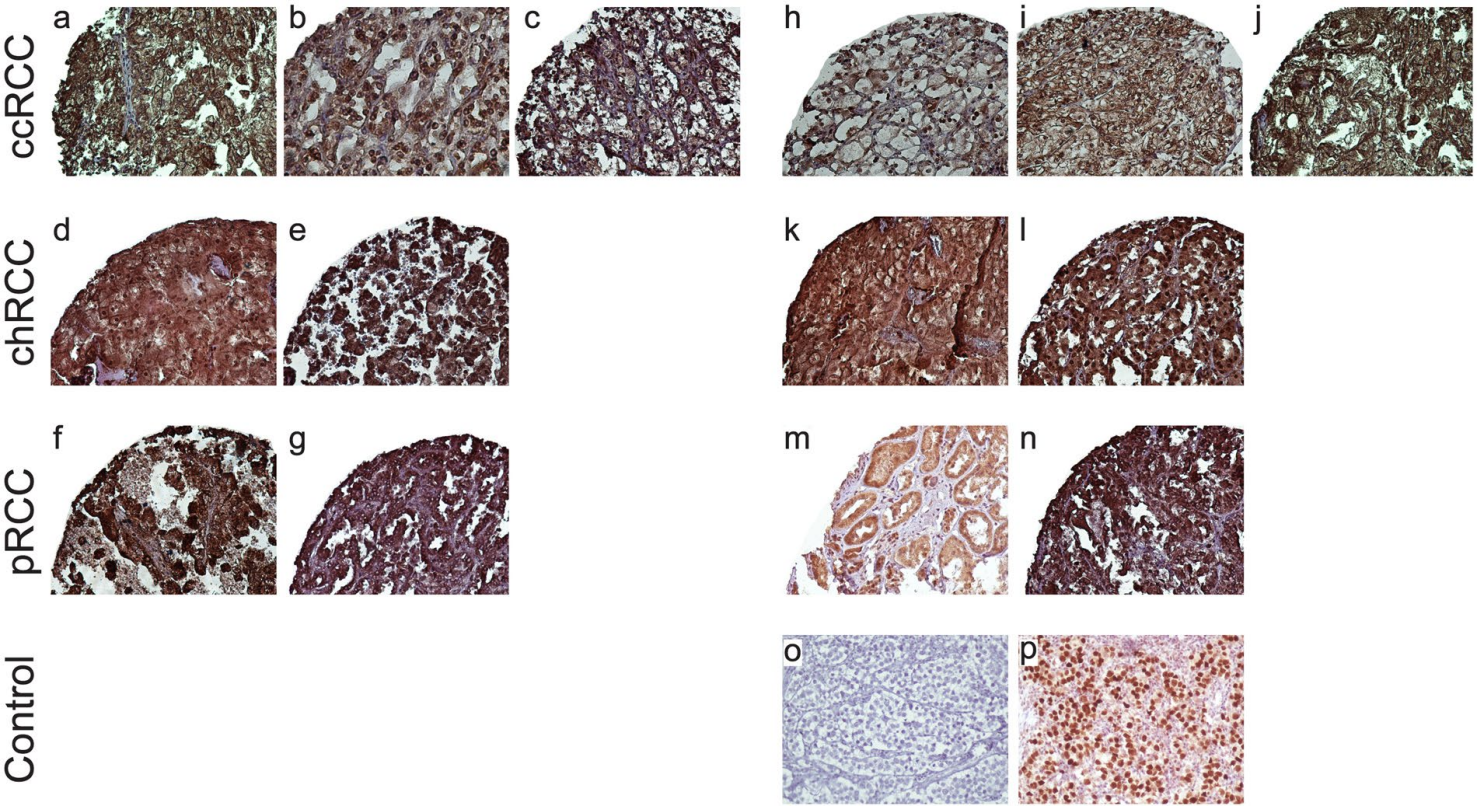
Immunohistochemical (IHC) Analysis of OCT4 Expression in Different Renal Cell Carcinoma (RCC) Samples. RCC samples expressed OCT4 at various levels. Nuclear expression of OCT4 in clear cell RCC at various levels: weak (**a**), moderate (**b**), and strong (**c**); chromophobe RCC: moderate (**d**), and strong (**e**); papillary RCC: weak (**f**), and moderate (**g**). Cytoplasmic expression of OCT4 in clear cell RCC at various levels is as follows: weak (**h**), moderate (**i**), and strong (**j**), chromophobe RCC: moderate (**k**), and strong (**l**); and papillary RCC: moderate (**m**), and strong (**n**). IHC staining of seminoma tissue was presented as negative (**o**) and positive (**p**) controls. Figures are displayed with a magnification of 200x.

**Figure 2:**
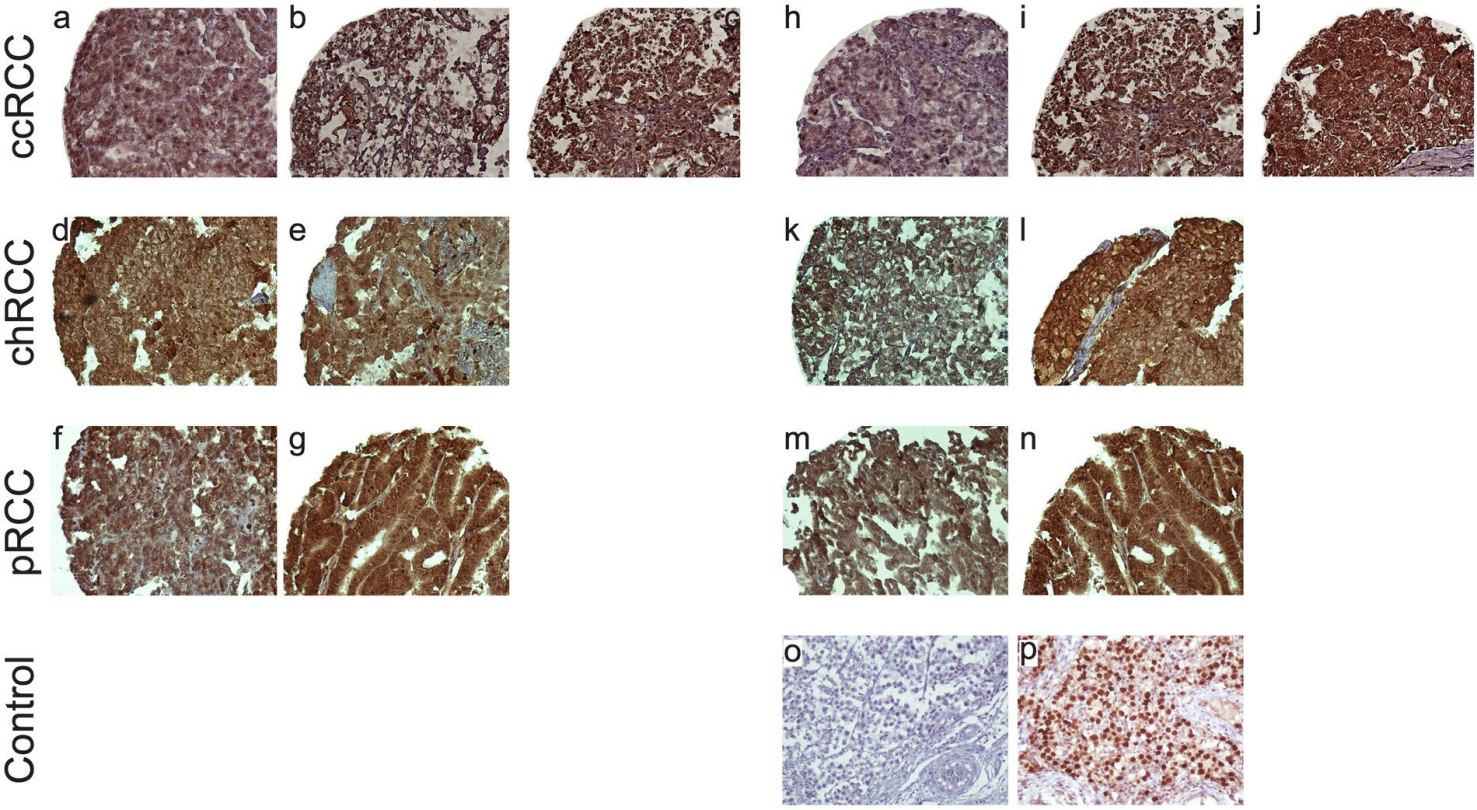
Immunohistochemical (IHC) Analysis of NANOG Expression in Different Renal Cell Carcinoma (RCC) Samples. RCC samples expressed NANOG at various levels. Nuclear expression of NANOG in clear cell RCC at various levels is as follows: weak (**a**), moderate (**b**), and strong (**c**); chromophobe RCC: weak (**d**), and moderate (**e**); and papillary RCC: weak (**f**), and strong (**g**). Cytoplasmic expression of NANOG in clear cell RCC at various levels is as follows: weak (**h**), moderate (**i**), and strong (**j**); chromophobe RCC: moderate (**k**), and strong (**l**); papillary RCC: moderate (**m**), and strong (**n**). IHC staining of seminoma tissue is presented as negative (**o**) and positive (**p**) controls. Figures are demonstrated with a magnification of 200x.

Analysis of TMA-based immunohistochemistry staining demonstrated that expression of OCT4 and NANOG was localized to the nucleus and cytoplasm of the tumor cells. In RCC, the respective nuclear and cytoplasmic expressions rate for OCT4 were 100% (186/186) and 95.2% (177/186), respectively, while the respective cytoplasmic and nuclear expressions rate for NANOG were 97.3% (181/186) and 82.8% (154/186), with varying levels of intensities. Immunohistochemical (IHC) analysis of OCT4 and NANOG expression in different RCC samples is illustrated in Figs 1 and 2.

The average nuclear and cytoplasmic expressions of OCT4 and NANOG (median H-score) in each subtype of RCC and the results of statistical tests are illustrated in Table 1. A statistically significant difference was found among the cytoplasmic expression of OCT4, the nuclear, and the cytoplasmic expression of NANOG in different RCC subtypes (*P* < 0.001). Also, a statistically significant difference was observed between the cytoplasmic expression of OCT4 in RCC different grades ((III/IV vs. I/II)), (*P* = 0.022).

**Table 1:** Average OCT4 and NANOG expressions (H-Score) in ccRCC, chRCC and pRCC tissues.

			ccRCC	ChRCC	pRCC	*P* value^a^	*P* value^b^	*P* value^c^	*P* value^d^
OCT4 Expression (*H*- Score)	Nuclear	Median (Q1, Q3)	200 (160, 240)	210 (200, 300)	200 (180, 240)	0.169	—	—	—
	Cytoplasmic	Median (Q1, Q3)	200 (200, 300)	300 (300, 300)	300 (300, 300)	**<*0***.***001***	**<*0***.***001***	**<*0***.***001***	0.568
NANOG Expression (*H*- Score)	Nuclear	Median (Q1, Q3)	100 (30, 200)	200 (100, 200)	200 (100, 300)	***<0***.***001***	***0***.***001***	***0***.***004***	0.899
	Cytoplasmic	Median (Q1, Q3)	200 (140, 300)	300 (300, 300)	300 (200, 300)	**<*0***.***001***	**<*0***.***001***	***0***.***001***	0.241

(Q1: the first quartile, Q3: the third quartile).

^a^Comparion between RCC subtypes using Kruskal-Wallis test.

Comparisons between RCC subtypes Mann-Whitney U test based on Bonferroni adjustment:

^b^Comparion between ccRCC and ChRCC subtypes.

^c^Comparion between ccRCC and pRCC subtypes.

^d^Comparion between ChRCC and pRCC subtypes.

Values in bold are statistically significant.

ccRCC indicates clear cell Renal Cell Carcinoma; ChRCC, chromophob Renal Cell Carcinoma; pRCC, papillary Renal Cell Carcinoma.

### Association of OCT4 and NANOG Expressions with Clinicopathologic Parameters in RCC

Association of OCT4 and NANOG expression with clinicopathologic characteristics in RCC is summarized in Table 2.

**Table 2:** Association of Nuclear expression (*H*-Score) of OCT4 and also nuclear and cytoplasmic expression (*H*-Score) of NANOG and clinical characteristics in RCC patients.

		Expression of OCT4			Expression of NANOG					
		Nuclear *H-Score* (N %)		*P value	Nuclear *H-Score* (N %)		*P value	Cytoplasmic *H-Score* N %)		*P value
Patient and Tumor Charachteristics	Total No. Cases 186 (N %)	Low (≤200)	*High* (200<)		Low (≤160)	*High* (160<)		Low (≤200)	*High* (200<)	
**Age (y)**										
≤55.2	87 (46.8)	55 (30.2)	29 (15.9)	0.644	40 (23.3)	41 (23.8)	0.359	42 (23.2)	42 (23.2)	0.882
>55.2	99 (53.2)	60 (33.0)	38 (20.9)		52 (30.2)	39 (22.7)		50 (27.6)	47 (26.0)	
**Sex**										
Male	130 (69.9)	54 (29.0)	76 (40.9)	1	58 (33.7)	63 (36.6)	503	67 (37.0)	60 (33.1)	0.516
Female	56 (30.1)	23 (12.4)	33 (17.7)		21 (12.2)	30 (17.4)		25 (13.8)	29 (16.0)	
**RCC subtypes**										
ccRCC	126 (67.7)	80 (44.0)	43 (23.6)		61 (35.5)	55 (32.0)	***0***.***041***	81 (44.8)	45 (29.4)	***<0***.***001***
ChRCC	29 (15.6)	13 (7.1)	15 (8.2)	0.113	9 (5.2)	18 (10.5)		3 (1.7)	26 (14.4)	
pRCC	31 (16.7)	22 (12.1)	9 (4.9)		9 (5.2)	20 (11.6)		8 (4.4)	18 (9.9)	
**Tumor size** (**cm)**										
<4	35 (18.8)	24 (13.2)	11 (6.0)		19 (11.0)	12 (7.0)		22 (12.2)	13 (7.2)	
04-Jul	65 (34.9)	43 (23.6)	2 (11.0)	0.182	31 (18.0)	29 (16.9)	0.748	28 (15.5)	34 (18.8)	0.307
07-Oct	44 (23.7)	28 (15.4)	15 (8.2)		23 (13.4)	19 (11.0)		23 (12.7)	19 (10.5)	
>10	42 (22.6)	20 (11.0)	21 (11.5)		19 (11.0)	20 (11.6)		19 (10.5)	23 (12.7)	
**Primary tumor** (**PT) Stage**										
I/II	75 (40.4)	50 (27.6)	22 (12.2)	0.159	35 (20.5)	29 (17.0)	0.874	38 (21.1)	34 (18.9)	0.651
III/IV	111 (59.6)	64 (35.4)	45 (24.9)		56 (37.7)	51 (29.8)		53 (29.4)	55 (30.6)	
**Histological Grade**										
I/II	94 (50.5)	65 (42.2)	27 (17.5)	0.169	49 (33.8)	37 (25.5)	1	44 (27.8)	49 (31.0)	1.000
III/IV	63 (33.9)	37 (24.0)	25 (16.2)		33 (22.8)	26 (17.9)		34 (21.5)	31 (19.6)	

*Significances are based on Pearson Chi-square test.

Values in bold are statistically significant.

ccRCC indicates clear cell Renal Cell Carcinoma; chRCC, chromophob Renal Cell Carcinoma and pRCC, papillary Renal Cell Carcinoma.

A statistically significant difference was observed between the nuclear and the cytoplasmic expression of NANOG in different RCC subtypes (*P*-values < 0.001 and <0.001, respectively). No other correlations were found between OCT and NANOG expressions and clinicopathologic characteristics.

### Combined Analysis of OCT4/NANOG Populations

Dual staining of markers is the best method to present concurrent expression of markers on the same cells, as both OCT4 and NANOG markers are expressed on the nucleus and cytoplasm; however, we were unable to perform double staining with matched antibodies. Therefore, immunohistochemistry was performed on the serial sections of RCC TMA blocks to show the expression of these markers in the best possible way. A significant reciprocal correlation was observed between expression of OCT4 and NANOG (Spearman correlation coefficient, 0.362; *P* < 0.001); thus, we explored the cumulative staining patterns of these markers and their association with clinicopathologic parameters in different subtypes of renal cancer. The expression levels of OCT4 and NANOG were divided into 2 categories in 3 conditions. Next, we examined the association between nuclear OCT4 and nuclear and cytoplasmic NANOG expressions with clinicopathologic parameters (Table 3).

**Table 3:** Association between the OCT4 and NANOG phenotypes with clinicopathologic parameters.

OCT4/NANOG Phenotypes										
Patients and Tumor Characteristics	Total No.Cases 186 (N %)	Condition1		*P value	Condition2		*P value	Condition3		*P value
		Group1	Group2		Group1	Group2		Group1	Group2	
		OCT4Nuclear^*high*^/NANOG-Nuclear^*high*^ or OCT4Nuclear^*high*^/NANOG-Cytoplasmic^*high*^	Other phenotypes		OCT4-Nuclear^*high*^/NANOG Nuclear^*high*^	Other phenotypes		OCT4Nuclear^*high*^/NANOG-Cytoplasmic^*high*^	Other phenotypes	
**RCC subtypes**										
ccRCC	126 (67.7)	57 (30.6)	69 (37.1)	**0.012**	34 (18.3)	92 (49.5)	0.116	53 (28.5)	73 (39.2)	**0.004**
ChRCC	29 (15.6)	22 (11.8)	7 (3.8)		13 (7.0)	16 (8.6)		22 (11.8)	7 (3.8)	
pRCC	31 (16.7)	16 (8.6)	15 (8.1)		12 (6.5)	19 (10.2)		14 (7.5)	17 (9.1)	
**Tumor size (cm)**										
<4	35 (18.8)	14 (7.5)	21 (11.3)	**0.016**	9 (4.8)	26 (14.0)	0.503	14 (7.5)	21 (11.3)	**0.009**
04-Jul	65 (34.9)	28 (15.1)	37 (19.9)		18 (9.7)	47 (25.3)		24 (12.9)	41 (22.0)	
07-Oct	44 (23.7)	23 (12.4)	21 (11.3)		15 (8.1)	27 (14.5)		22 (11.8)	22 (11.8)	
>10	42 (22.6)	30 (16.1)	12 (6.5)		59 (31.7)	27 (14.7)		29 (15.6)	13 (7.0)	
**Primary tumor (PT) Stage**										
I/II	74 (39.8)	35 (18.9)	38 (20.5)	**0.547**	21 (11.4)	52 (28.1)	0.52	31 (16.8)	42 (22.7)	0.231
III/IV	112 (60.2)	60 (32.4)	52 (28.1)		38 (20.5)	74 (40.0)		58 (31.4)	54 (29.2)	
**Histological Grade**										
I/II	94 (50.5)	38 (24.2)	56 (35.7)	0.7	23 (14.6)	71 (45.2)	0.111	34 (21.7)	60 (38.2)	0.05
III/IV	63 (33.9)	35 (22.3)	28 (17.8)		23 (14.6)	40 (25.5)		33 (21.0)	30 (19.1)	
**Microvascular invasion(MVI)**										
Positive	38 (20.4)	25 (14.2)	13 (7.4)	**0.03**	16 (9.1)	22 (12.5)	0.345	24 (13.6)	14 (8.0)	0.076
Negative	132 (71.0)	61 (34.7)	71 (40.3)		39 (22.2)	93 (52.8)		58 (33.0)	74 (42.0)	

*Significances are based on Pearson Chi-square test.

Values in bold are statistically significant.

ccRCC indicates clear cell Renal Cell Carcinoma; chRCC, chromophob Renal Cell Carcinoma; pRCC, papillary Renal Cell Carcinoma.

### Prognostic Significance of OCT4 and NANOG Expressions

**Figure 3:**
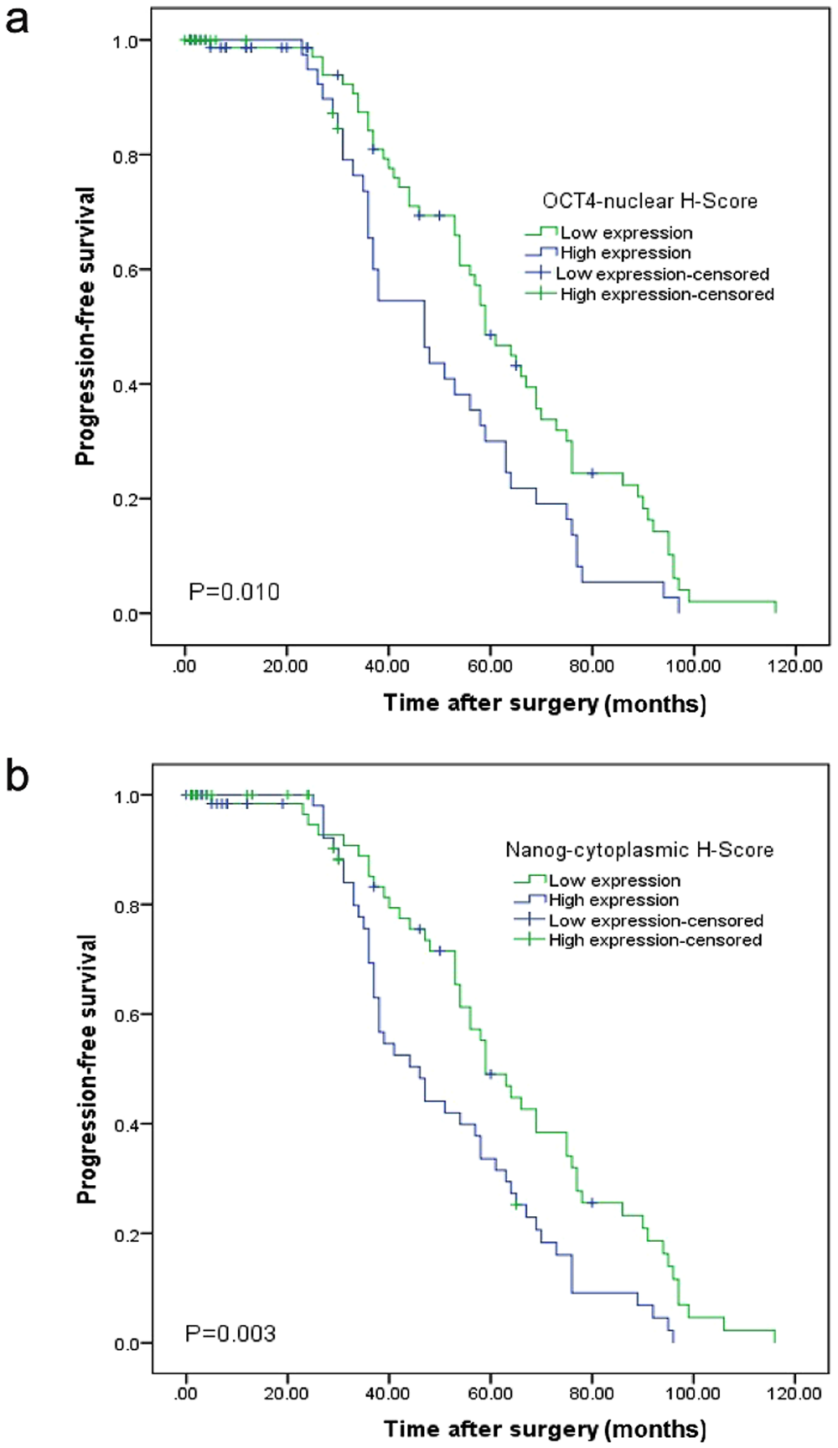
Correlation Between OCT4 and NANOG Expressions and Survival Rates in Patients with Renal Cell Carcinoma. Progression-free survival (PFS) with nuclear OCT4 (**a**) and cytoplasmic NANOG (**b**) expressions (Nuclear and cytoplasmic expressions were grouped into low- vs. moderate- vs. high-expression levels).

The 5-year DSS and PFS survival rates were 82.0% and 49.0% in low nuclear OCT4 expression, and 84.0% and 30.0% in high nuclear OCT4 expression. OCT4 nuclear overexpression was associated with shorter PFS survival than the low expression group (*P* = 0.010) (Fig. 3a).

In the survival analysis of NANOG nuclear expression, the 5-year DSS and PFS survival rates were 79.0% and 40.0% in cases with low nuclear NANOG expression, and 86.0% and 41.0% in cases with high nuclear NANOG expression. The 5-year DSS and PFS survival rates were 82.0% and 49.0% in cases with low cytoplasmic NANOG expression, and 82.0% and 34.0% in cases with high cytoplasmic NANOG expression. Cytoplasmic overexpression of NANOG was associated with shorter PFS survival rate than the low expression group (*P* = 0.003) (Fig. 3b).

**Figure 4:**
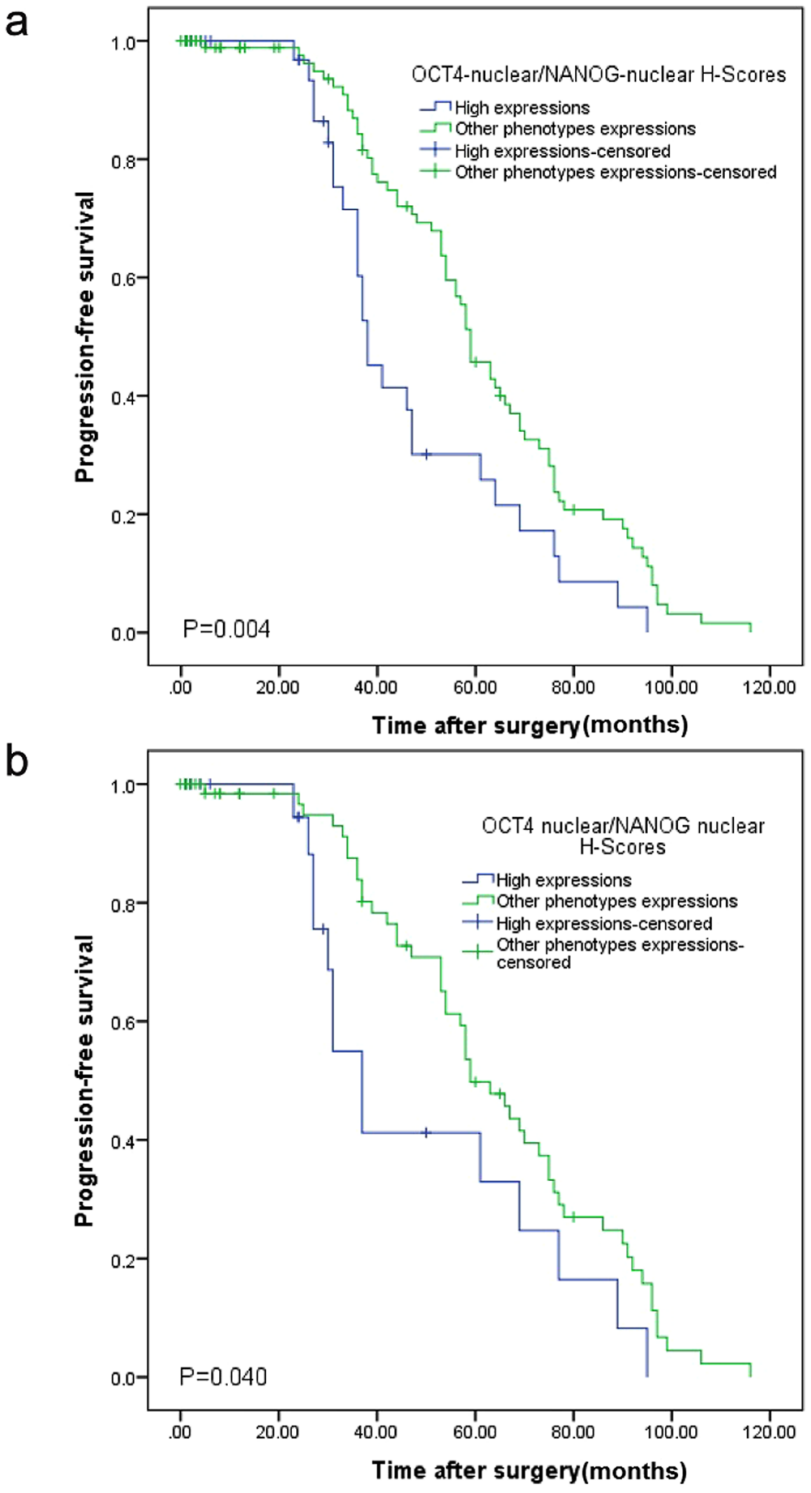
Influence of Coexpression of OCT4 and NANOG on Progression-Free Survival (PFS). PFS in renal cell carcinoma (RCC) patients (**a**), and PFS in clear cell renal cell carcinoma (ccRCC) patients (**b**). Patients with RCC (**a**) and ccRCC (**b**) showed worse PFS with high coexpression of nuclear OCT4 and nuclear NANOG in tumors.

Then, the survival rate of patients with coexpression of OCT4 and NANOG (OCT4-nuclear^*high*^/NANOG-nuclear^*high*^ and OCT4-nuclear^*high*^/NANOG-cytoplasmic^*high*^) was compared with that of other phenotypes. Higher nuclear expressions of OCT and NANOG were associated with worse progression-free survival than other phenotypes (*P* = 0.004) (Fig. 4a).

Cox proportional univariate and multivariable analysis of relationships between prognostic variables and survival are presented in Table 4. Fuhrman grade and Tumor size were independent risk factor affecting the DSS of patients with RCC in multivariable analysis, with hazard ratios of 2.83 and 1.68 and *p*-values of 0.020 and 0.024, respectively. Clinical stage and cytoplasmic NANOG expression showed independent poor PFS, with hazard ratios of 2.41 and 1.58 and *p*-values of <0.001 and 0.041, respectively.

**Table 4:** Univariate and multivariable analysis of disease free survival (DSS) and progression-free survival (PFS) in patients with renal cell carcinoma (RCC).

Feature	DSS				PFS			
	Univariate		Multiivariable		Univariate		Multiivariable	
	HR (95% CI)	*P* value	HR (95% CI)	*P* value	HR (95% CI)	*P* value	HR (95% CI)	*P* value
**Age**								
(>55.2 vs. ≤55.2)	1.15 (0.46–2.87)	0.759	—	—	1.68 (1.00–2.79)	0.046	—	—
**Sex**								
(male vs. female)	0.91 (0.33–2.55)	0.872	—	—	0.93 (0.53–1.63)	0.804	—	—
**Clinical stage**								
(III/IV vs. I/II)	2.25 (0.85–5.97)	0.103	—	—	2.40 (1.34–4.30)	**0.003**	2.41 (1.48–3.91)	**<0.001**
**Fuhrman grade**								
(III/IV vs. I/II)	3.70 (1.47–9.30)	**0.005**	2.83 (1.17–6.81)	**0.020**	1.73 (0.97–3.08)	0.063	—	—
**Tumor size**	1.22 (1.08–1.38)	**0.001**	1.68 (1.07–2.66)	**0.024**	1.04 (0.96–1.12)	0.354	—	—
**MVI**								
(Present vs. Absent)	0.39 (0.14–1.04)	0.062	—	—	1.05 (0.94–1.16)	0.354	—	—
**Nuclear OCT4 Expression**								
High vs. low	0.62 (0.20–1.88)		0.399	—	—	1.60 (0.94–2.72)	0.079	—
**Nuclear NANOG Expression**								
High vs. low	1.00 (0.37–2.68)	0.986	—	—	1.38 (0.89–2.12)	0.141	—	—
**Cytoplasmic NANOG Expression**								
High vs. low	1.00 (0.39–2.54)	0.996	—	—	1.86 (1.22–2.83)	**0.004**	1.58 (1.02–2.45)	**0.041**
**OCT4 nuclear/NANOG nuclear**								
High vs. Other phenotypes	0.84 (0.30–2.35)	0.752	—	—	0.51 (0.32–0.82)	**0.005**	0.55 (0.34–0.90)	**0.018**
**OCT4 nuclear/NANOG cytoplasmic**								
High vs. Other phenotypes	0.83 (0.33–2.08)	0.706	—	—	0.70 (0.46–1.64)	0.096	—	—

Abbreviations: HR hazard ratio; CI confidence interval MVI; Microvascular invasion.

Values in bold are statistically significant.

The variables with P value less than 0.2 were included in multivariable analyses.

### Association of OCT4 and NANOG Expressions with Survival Outcomes in ccRCC

Due to the limited number of occurred events in pRCC and ChRCC subtypes, DSS, PFS, and Cox proportional univariate and multivariable analyses were performed only for ccRCC patients. In Kaplan-Meier survival analysis, ccRCC patients, whose tumors expressed higher nuclear levels of OCT4 and NANOG, showed significantly poorer PFS than other phenotypes expressions (*P* = 0.040). (Fig. 4b). Tumor size and Fuhrman grade were the only significant risk factors affecting the DSS of patients with ccRCC in multivariable Cox proportional analysis, with hazard ratios of 1.22 and 1.47 and *p*-values of 0.001 and 0.005, respectively. Stage was the only significant risk factor affecting the PFS of patients with ccRCC in multivariable Cox proportional analysis, with hazard ratios of 2.35and *p*-value of 0.004.

## Discussion

Many renal cancer patients experience disease recurrence after receiving combined treatments or immunotherapy due to the permanence of CSCs^29^. The expression of OCT4 or NANOG has been reported in the CSCs and been associated with a more aggressive tumor phenotype^30^. In the present study, for the first time, the expression levels of both OCT4 and NANOG were investigated in a well- characterized series of 186 tissues samples from 3 main subtypes of RCC; moreover, the impact of OCT4 and NANOG coexpression in RCC prognosis was evaluated.

Similar to previous reports, we found both nuclear and cytoplasmic expression pattern of OCT4 and NANOG in RCC samples^31,32^. This staining pattern of OCT4 may arise from the presence of an OCT4 isoform. OCT4 is known to have 2 isoforms, OCTA and OCTB. OCT4A is observed in the nucleus and OCT4B in the cytoplasm in prostate and cervical cancers^32,33^. Shao-Wen L.*et al*. demonstrated that both OCT4A and OCT4B are highly expressed at different subcellular locations. OCT4A appears to be responsible for stemness of CSCs and triggers carcinogenesis, while OCT4B promotes tumor growth by the regulation of angiogenesis, apoptosis, and epithelial to mesenchymal transition (EMT)^33^.

Our immunohistochemical analysis revealed that 100% of RCC samples were positive for Oct-4, 98% were positive for NANOG, 31.6% for OCT4-nuclear^*high*^/NANOG-nuclear^*high*^, and 47.6% were OCT4-nuclear^*high*^/NANOG cytoplasmic^*high*^ phenotypes. Positive OCT4 or NANOG expression in renal cancer was not associated with known prognostic factors, such as clinical stage, or tumor size; however, it was significantly associated with histological subtype. Similar to our result, Bin YU *et al*. reported that the positive expression rate of OCT4 was not significantly correlated with T stage, but they did not refer to the expression pattern of OCT 4 in RCC samples. They demonstrated that RCC patients with high OCT4 and NANOG expressions in tumor tissues had significantly lower survival time and metastasis-free survival rate^31^.

More importantly, the coexpression of OCT4 and NANOG in renal cancer was significantly associated with RCC subtypes, tumor size, and MVI in our study. The significant difference in nuclear OCT4 and nucleocytoplasmic NANOG expressions between ccRCC, compared with the chRCC and pRCC tumor samples, indicates that OCT4 and NANOG may act as a diagnostic markers to distinguish ccRCC from chRCC and pRCC subtypes.

It has been confirmed that ccRCC is more aggressive and associated with poorer prognosis than papillary and chromophobe RCC subtypes^34^. Most ccRCCs cases have deletions in the short arm of Chromosome 3, which are highly specific for ccRCC, and are not observed in any other RCC subtypes. These deletions result in the loss of specific small regions including the von Hippel–Lindau (VHL) gene^35^. On the other hand, VHL mutation induces overexpression of the hypoxia-inducible factor (HIF).The activation of vascular endothelial growth factor (VEGF) by HIF-1 has been observed as a notable mechanism involved in metastasis and hypoxia-induced angiogenesis^29,36^. HIF-2α has oncogenic function in ccRCC and promotes c-Myc, a vital oncogenic stem cell factor activity in pVHL −/− ccRCC, inhibits p53 activity, and decreases subsequent p53-induced apoptosis upon γ-radiation, resulting in radio resistance, a significant feature of CSCs^37^. It is also involved in the expansion of CXCR4-positive cancer stem-like cells in RCC^38^. Previous studies have shown that hypoxic regulation of 2 stem cell factors, OCT4 and c-Myc, may powerfully impact CSC formation from cells expressing HIF-2α^37,39^. Like c-Myc and Oct4, NANOG, Sox2, and Klf4 are HIF-2α-dependent^40^. This mechanism may also occur in ccRCC. Further investigations are required to find a relationship among increased expressions of OCT4 and NANOG, hypoxia, HIF-2α expression, and CSC induction in ccRCC.

In this study, tumors with high nuclear expression of OCT4 or cytoplasmic NANOG expression had worse PFS survival. Bin Y. *et al*., in agreement with our study, demonstrated that RCC patients with higher expression of OCT4 and NANOG in tumor tissues had significantly lower survival time and metastasis-free survival rate^31^. Interestingly, cytoplasmic NANOG expression was an independent prognostic factor for poor PFS in RCC patients, which was a novel finding of our study, raising the possibility of its utility as a prognostic biomarker for RCC. The follow-up period was not sufficiently long to allow definitive conclusions on some prognostic issues.

Overall, many preliminary studies have supported the fact that increased expression of OCT4 and NANOG correlates with poor prognosis, adverse clinicopathologic features, and survival time^18,19^. The expression of OCT4 has further been shown in human cancer stem-like cells, implicating its involvement in tumorigenesis and self-renewal through activating its downstream target genes^13^. Moreover, T. Hu *et al*. suggested that a reduction in OCT4 expression in lung CSCs induces the inhibition of tumor growth and apoptosis^41^. On the other hand, recent studies have shown that increased NANOG expression was observed in enriched CSCs. The discoveries of downstream regulatory pathways, directly or indirectly mediated by NANOG, indicate that NANOG regulates several aspects of cancer development, such as self-renewal, tumor cell proliferation, motility, EMT, escape from immune system, and drug resistance, which are all defined features for CSCs^42^.

Similar to our observations, coexpression of OCT4 and NANOG was found to be significantly associated with tumor aggressiveness and poor prognosis of several malignances including breast and lung cancers and glioma^16,43,44^. Importantly, a direct link was also found between OCT4 and NANOG. Together, these 2 markers can guide a cascade of pathways, which lead to the self-renewal and pluripotency characteristics of ESCs and contribute to tumor aggressiveness, poor outcome, and CSC characteristics^8,45^. Some recent studies have shown that ectopic coexpression of OCT4 and NANOG empowers cells with CSC properties including extensive proliferation, self-renewal, high tumorigenic capacity, and drug resistance. OCT4 and NANOG significantly influence EMT change and contribute to tumor migration, invasion, and metastasis *in vitro* and *in vivo*^16,20^. On the other hand, Wang D. *et al*. demonstrated that knockdown of both OCT4 and NANOG expressions inhibit spontaneous changes in the expression of EMT-related genes and the migration of breast CSC *in vitro*^44^.

Evidence shows a direct link between the EMT and CSCs, both of which are involved in the generation of invasive cells and formation of distant metastases^16^. The majority of cancer cells in a tumor are non-tumorigenic, and therapy targeting these cells may cause tumor regression. However, if therapy fails to target the tumorigenic CSCs, these cells would persist after treatment and could regenerate the tumor, causing tumor recurrence or relapse. Therefore, eradication of cancers requires the elimination of CSCs. Advanced strategies that specifically target CSCs without damaging normal cells are urgently needed to improve current therapeutic treatments^42^.

Our data showed that RCC and ccRCC patients with higher coexpression of nuclear OCT4 and NANOG had worse PFS. Moreover, a significant difference was found in OCT4 and NANOG coexpression in ccRCC due to its worst prognosis compared with other RCC subtypes. Although our findings suggest that both nuclear expression of OCT4 and nucleocytoplasmic expressions of NANOG have implications in RCC prognosis, conducting further studies with larger sample size and prolonged follow-up time is highly recommended.

## Conclusions

Our findings further suggest that OCT4 and NANOG coexpression may be valuable biomarkers in predicting the outcome of patients with renal cancer, especially in the ccRCC subtype. In the present study, a significant positive relationship was found between high expression of OCT4 and NANOG. Based on these findings, we suggest that there might be a positive involvement of OCT4/ NANOG, signaling pathways in tumor invasion and progression of RCC. Thus, it can be concluded that increased cytoplasmic expression of NANOG is an independent prognostic predictor in patients with RCC. Data revealed that abnormal elevated expression levels of key stemness factors, such as OCT4 and NANOG, in several types of CSCs, the role of these CSC markers in generation and maintenance of renal CSC, and the importance and therapeutic potential of targeting these stemness regulators in RCC have attracted the attention of the researchers. In addition, it would be helpful to gain knowledge about the pathological analysis of the subcellular localization of different OCT4 isoforms and the relationship of OCT4 and NANOG with renal CSCs properties, which will provide important clues to the diagnosis and prognosis of renal cancer and raise exciting questions for future experiments.

## References

[1] Jemal A (2009). Cancer statistics, 2009. CA: a cancer journal for clinicians.

[2] Ingels A (2014). Preclinical trial of a new dual mTOR inhibitor, MLN0128, using renal cell carcinoma tumorgrafts. International journal of cancer.

[3] Reya T, Morrison SJ, Clarke MF, Weissman IL (2001). Stem cells, cancer, and cancer stem cells. nature.

[4] Mani SA (2008). The epithelial-mesenchymal transition generates cells with properties of stem cells. Cell.

[5] Shan J (2012). Nanog regulates self‐renewal of cancer stem cells through the insulin‐like growth factor pathway in human hepatocellular carcinoma. Hepatology.

[6] Boiani M, Schöler HR (2005). Regulatory networks in embryo-derived pluripotent stem cells. Nature reviews Molecular cell biology.

[7] Park I-H (2008). Reprogramming of human somatic cells to pluripotency with defined factors. nature.

[8] Kumar SM (2012). Acquired cancer stem cell phenotypes through Oct4-mediated dedifferentiation. Oncogene.

[9] Ezeh UI, Turek PJ, Reijo RA, Clark AT (2005). Human embryonic stem cell genes OCT4, NANOG, STELLAR, and GDF3 are expressed in both seminoma and breast carcinoma. Cancer.

[10] Yu J (2007). Induced pluripotent stem cell lines derived from human somatic cells. science.

[11] Okita K, Ichisaka T, Yamanaka S (2007). Generation of germline-competent induced pluripotent stem cells. nature.

[12] Scholer HR, Ruppert S, Suzuki N, Chowdhury K, Gruss P (1990). New type of POU domain in germ line-specific protein Oct-4. Nature.

[13] Ponti D (2005). Isolation and *in vitro* propagation of tumorigenic breast cancer cells with stem/progenitor cell properties. Cancer research.

[14] Chambers I (2003). Functional expression cloning of Nanog, a pluripotency sustaining factor in embryonic stem cells. Cell.

[15] Feske S (2007). Calcium signalling in lymphocyte activation and disease. Nature Reviews Immunology.

[16] Chiou S-H (2010). Coexpression of Oct4 and Nanog enhances malignancy in lung adenocarcinoma by inducing cancer stem cell–like properties and epithelial–mesenchymal transdifferentiation. Cancer research.

[17] Wen J (2010). Oct4 and Nanog expression is associated with early stages of pancreatic carcinogenesis. Pancreas.

[18] Meng H-M (2010). Over-expression of Nanog predicts tumor progression and poor prognosis in colorectal cancer. Cancer biology & therapy.

[19] Jeter CR (2009). Functional evidence that the self‐renewal gene NANOG regulates human tumor development. Stem cells.

[20] Yin X (2015). Coexpression of gene Oct4 and Nanog initiates stem cell characteristics in hepatocellular carcinoma and promotes epithelial-mesenchymal transition through activation of Stat3/Snail signaling. Journal of hematology & oncology.

[21] Srigley JR (2013). The International Society of Urological Pathology (ISUP) vancouver classification of renal neoplasia. The American journal of surgical pathology.

[22] Rasti A (2017). Reduced expression of CXCR4, a novel renal cancer stem cell marker, is associated with high-grade renal cell carcinoma. Journal of Cancer Research and Clinical Oncology.

[23] Roudi R, Korourian A, Shariftabrizi A, Madjd Z (2015). Differential expression of cancer stem cell markers ALDH1 and CD133 in various lung cancer subtypes. Cancer investigation.

[24] Erfani E (2015). Comparative expression analysis of putative cancer stem cell markers CD44 and ALDH1A1 in various skin cancer subtypes. The International journal of biological markers.

[25] Madjd Z, Ramezani B, Molanae S, Asadi-Lari M (2012). High expression of stem cell marker ALDH1 is associated with reduced BRCA1 in invasive breast carcinomas. Asian Pacific Journal of Cancer Prevention.

[26] Mohsenzadegan M (2013). Reduced expression of NGEP is associated with high-grade prostate cancers: a tissue microarray analysis. Cancer Immunology, Immunotherapy.

[27] McCarty K, Miller L, Cox E, Konrath J, McCarty Sr K (1985). Estrogen receptor analyses. Correlation of biochemical and immunohistochemical methods using monoclonal antireceptor antibodies. Archives of pathology & laboratory medicine.

[28] Turun S, Banghua L, Zheng S, Wei Q (2012). Is tumor size a reliable predictor of histopathological characteristics of renal cell carcinoma?. Urology annals.

[29] Matak D (2015). Biology of renal tumour cancer stem cells applied in medicine. Contemporary Oncology.

[30] Gidekel S, Pizov G, Bergman Y, Pikarsky E (2003). Oct-3/4 is a dose-dependent oncogenic fate determinant. Cancer cell.

[31] Yu B (2016). Expressions of stem cell transcription factors Nanog and Oct4 in renal cell carcinoma tissues and clinical significance. Artificial cells, nanomedicine, and biotechnology.

[32] de Resende MF (2013). Prognostication of OCT4 isoform expression in prostate cancer. Tumor Biology.

[33] Li S-W (2015). The differential expression of OCT4 isoforms in cervical carcinoma. PloS one.

[34] Lau WK, Cheville JC, Blute ML, Weaver AL, Zincke H (2002). Prognostic features of pathologic stage T1 renal cell carcinoma after radical nephrectomy. Urology.

[35] Hansel D (2006). Genetic alterations and histopathologic findings in familial renal cell carcinoma. Histology and histopathology.

[36] Schneider BP, Sledge GW (2007). Drug insight: VEGF as a therapeutic target for breast cancer. Nature Reviews. Clinical Oncology.

[37] Gordan JD, Bertout JA, Hu C-J, Diehl JA, Simon MC (2007). HIF-2α promotes hypoxic cell proliferation by enhancing c-myc transcriptional activity. Cancer cell.

[38] Micucci C, Matacchione G, Valli D, Orciari S, Catalano A (2015). HIF2α is involved in the expansion of CXCR4-positive cancer stem-like cells in renal cell carcinoma. British journal of cancer.

[39] Covello KL (2006). HIF-2α regulates Oct-4: effects of hypoxia on stem cell function, embryonic development, and tumor growth. Genes & development.

[40] Hamaï A, Codogno P, Mehrpour M (2014). Cancer stem cells and autophagy: facts and perspectives. Journal of cancer stem cell research.

[41] Hu T (2008). Octamer 4 small interfering RNA results in cancer stem cell–like cell apoptosis. Cancer research.

[42] Wang, M.-L., Chiou, S.-H. & Wu, C.-W. Targeting cancer stem cells: emerging role of Nanog transcription factor. OncoTargets & Therapy **6** (2013). 10.2147/OTT.S38114 PMC3772775 24043946

[43] Guo Y (2011). Expression profile of embryonic stem cell‐associated genes Oct4, Sox2 and Nanog in human gliomas. Histopathology.

[44] Wang D (2014). Oct-4 and Nanog promote the epithelial-mesenchymal transition of breast cancer stem cells and are associated with poor prognosis in breast cancer patients. Oncotarget.

[45] Loh Y-H (2006). The Oct4 and Nanog transcription network regulates pluripotency in mouse embryonic stem cells. Nature genetics.

